# A Multicentric, Prospective, Observational, Single-Arm Registry Study to Assess the Clinical Safety and Effectiveness of Thrombophob Ointment (Heparin Sodium + Benzyl Nicotinate) in Indian Patients With Thrombophlebitis

**DOI:** 10.7759/cureus.54436

**Published:** 2024-02-19

**Authors:** Harsha Somavarapu, Kumar Kamala Kant Rakesh, Gyan Roy, Vinod Kumar Navkar, Nimitha Pinto, Sameer Muchhala, Pavankumar M Daultani, Ravindra Mittal

**Affiliations:** 1 Orthopaedic and Spine Surgery, Tejaswi Ortho & Trauma Hospital, Tenali, IND; 2 General and Laparoscopic Surgery, Orchid Hospital, Patna, IND; 3 Surgery, Roy Clinic, Delhi, IND; 4 General Surgery, Navkar Day Care Center, Seoni, IND; 5 Medical Affairs, Zydus Healthcare Ltd., Mumbai, IND; 6 New Product Development, Zydus Healthcare Ltd., Ahmedabad, IND

**Keywords:** benzyl nicotinate, vas scores, heparin sodium, thrombophob ointment, thrombophlebitis

## Abstract

Purpose

Thrombophlebitis is a frequent intravenous (IV) therapy consequence. Topical heparin for seven days is used as a treatment for thrombophlebitis. This study was performed to evaluate the clinical safety and effectiveness of the combination of heparin sodium & benzyl nicotinate (Thrombophob Ointment, manufactured by Zydus Healthcare Ltd., Ahmedabad, India) in thrombophlebitis patients in India.

Methods

A study carried out by 118 Indian doctors examined 2002 thrombophlebitis patients from 2016-2023, prescribing ointment containing heparin sodium and benzyl nicotinate. Patients were followed up on day three and day seven after starting the treatment, and safety and effectiveness were recorded, including adverse events.

Result

A total of 2002 patients were included in the study and males were predominant (58.15%). IV fluids (60.58%) were the leading cause of thrombophlebitis. The study found notable improvements in key markers of venous health over time. Compared to baseline, patients experienced significantly reduced severity of phlebitis, shorter venous lesion lengths, and lower pain and tenderness scores by both day 3 and day 7 (p<0.001 for all comparisons). Furthermore, these improvements continued between day 3 and day 7, indicating sustained positive effects (p<0.001 for all comparisons). After the application of the ointment, very few patients experienced adverse effects (0.25% on day three and 0.05% on day seven). Treatment effectiveness was excellent in 72% of patients, and treatment safety was excellent in 93% of patients.

Conclusion

The ointment containing heparin sodium and benzyl nicotinate was well tolerated and efficacious in the treatment of thrombophlebitis in Indian patients.

## Introduction

Intravenous (IV) cannulation is an important and common aspect of hospital practice for the administration of medications, nutrients, fluids, and blood products and for monitoring the hemodynamic status of a person [1]. Phlebitis is one of the common complications of IV therapy and 25% to 70% of patients receiving IV therapy develop phlebitis largely after 48 hours perioperatively [2,3]. Phlebitis is characterized by inflammation occurring inside a vein, while thrombosis is defined by the formation of a clot within a vein [4]. Phlebitis refers to the subjective clinical manifestation at an access site with symptoms: redness, pain, swelling, palpable venous cords, thrombosis, or streak formation. Infection or the formation of a thrombus can result from this condition. Symptoms typically emerge over a span of hours to days and generally subside within a few days to weeks [3,5].

There are three different types of phlebitis namely: mechanical, chemical, and infectious. Mechanical phlebitis occurs when a peripheral IV catheter is not secured properly, leading the catheter to change position within the vein [6]. Chemical phlebitis is caused by highly vesicant irritants such as drugs. Drug irritation was indicated as the most significant predictor of phlebitis such as antibiotics, blood products, and glucose-containing fluids [7]. Infectious or bacterial phlebitis occurs when an infectious agent is introduced into the peripheral IV catheter. Infectious phlebitis can be caused by contamination of the catheter tip anytime during IV insertion. Infectious phlebitis may also occur if a cannula is left in place longer than recommended by the Centre for Disease Control and Prevention (CDC) [1,7,8].

Superficial venous thrombophlebitis (SVT) is a prevalent condition where a thrombus forms in a superficial vein, close to the skin's surface. Research conducted in the emergency and surgical departments of a hospital revealed that 29.8% of cases involving peripheral IV cannulas are linked to phlebitis. The occurrence rate can reach up to 75%. Although the exact causes are often unclear, it is believed that IV catheters may trigger endothelial damage and inflammation, subsequently resulting in venous thrombosis [9].

The CDC recommends rotating the IV cannula every 72 to 96 hours to reduce the risk of infection and patient discomfort associated with phlebitis [10]. Phlebitis causes pain and sepsis and may need additional diagnostic investigations and treatments. These may lead to the increased duration of hospitalization, patient stress level, and financial burden as well as increasing staff workload. Treatment is needed for the local symptoms and to prevent life-threatening systemic complications like deep venous thrombosis [11].

Generally, treatment for SVT involves applying topical heparin for a duration of seven days. Heparin, an anticoagulant, mainly works by preventing coagulation and halting the advancement of the condition, although it has minimal impact on clots that have already formed. Starting the application of prophylactic topical heparin from the first day of IV cannula insertion, before the onset of thrombophlebitis, could be more beneficial in averting or postponing the condition [12].

Heparin is an anticoagulant, while benzyl nicotinate is a vasodilator. Heparin is a linear glycosaminoglycan (GAG) and has been used as an anticoagulant for the treatment of thromboembolic conditions for many decades. Heparin functions by reversibly attaching to antithrombin III, increasing antithrombin III's ability to deactivate thrombin and factor Xa, which ultimately promotes fibrinolysis, whereas benzyl nicotinate induces vasodilatation and increases the body's capability to absorb local heparin. When combined topically, they dilate blood vessels and dissolve or stop blood clots from forming. This reduces inflammation or swelling of a vein caused by a blood clot in the superficial thrombophlebitis by increasing blood flow to the afflicted area and reducing the accompanying symptoms. Additionally, it is used to treat hematomas, or collections of blood under the skin, and bruises. It reduces the discomfort and edema associated with treating superficial thrombophlebitis by hastening the blood clot's disintegration. The combination of heparin sodium and benzyl nicotinate is generally safe to use with minimal or no side effects. However, over the last decade, there have been concerns about the tolerability of heparin [13]. The evidence about treating and preventing acute thrombophlebitis with different drug formulations is limited and of low quality in the medical, surgical, and anesthetic literature [13]. Thus, this study was conducted to assess the clinical safety & effectiveness of the combination of heparin sodium and benzyl nicotinate (Thrombophob Ointment, Zydus Healthcare Ltd., Ahmedabad, India) in Indian patients with thrombophlebitis.

## Materials and methods

Study design

This was a prospective, observational, multi-centric, single-arm registry study conducted by 118 doctors across India, on 2002 patients diagnosed with thrombophlebitis from April 2016 to April 2023 (seven years). Data for safety and effectiveness were collected from the doctors who prescribed Thrombophob ointment (Heparin Sodium IP 50 I.U. and Benzyl Nicotinate 2mg per gm of ointment) for the treatment of thrombophlebitis. This data was collected in data collection forms.

Study participants

Study participants were those who were diagnosed with thrombophlebitis and treated with Thrombophob ointment.

Study methodology

The patients who were prescribed Thrombophob Ointment for thrombophlebitis applied it over the affected areas 2-3 times per day as directed by the doctors. The participants were followed up by the doctor on day three and day seven and parameters of safety and effectiveness were recorded. Demographic and clinical data (cause of thrombophlebitis; duration of phlebitis before the treatment was initiated; details of treatment) of study participants were recorded by the doctors on day zero. The parameters of effectiveness like the length of the venous lesion; visual analog scale (VAS) score for pain (0-10); VAS score for tenderness (0-10); grade of phlebitis (on Phlebitis rating Scale) (Table 1) [14]; and any adverse events reported during the treatment were recorded on days three and seven of treatment given. Global assessment of effectiveness and tolerability was recorded at the end of the study (day seven) (Table 2) [15].

**Table 1:** Grade of phlebitis IV: Intravenous Source: Reference [14]

Grade	Stage	Observation
Grade 0	No sign of phlebitis	IV site appears healthy
Grade 1	Possibly first signs of phlebitis	One of the following is evident: Slight pain near IV site slight redness near the IV site
Grade 2	Early stage of phlebitis	Two of the following are evident: Pain at IV site redness swelling
Grade 3	Medium stage of phlebitis	All are evident: Pain along path of cannula redness swelling
Grade 4	Advance stage of phlebitis/thrombophlebitis	All are evident and extensive: Pain along path of cannula redness swelling palpable venous cord
Grade 5	Advanced stage of thrombophlebitis	All are evident and extensive: Pain along path of cannula redness swelling palpable venous cord Pyrexia

**Table 2:** Category of Physician’s Global Assessment of effectiveness and tolerability Source: Reference [15]

Global assessment of Effectiveness	
Excellent	Complete resolution of symptoms
Good	>50% reduction in length of venous lesion
Fair	<50% reduction in length of venous lesion
Poor	No response or increase in length of venous lesion
Global assessment of Tolerability	
Excellent	No adverse event reported
Good	Mild adverse event(s) reported which subsided with or without medication and did not necessitate stoppage of study medication
Fair	Moderate to severe adverse event(s) reported which subsided with or without medication and did not necessitate stoppage of study medication
Poor	Severe or serious adverse event(s) which necessitated stoppage of study medication

Effectiveness outcome

Effectiveness outcomes of the study were the mean difference in the grade of phlebitis, length of venous lesion, VAS score for pain, and VAS score for tenderness on day three and day seven from baseline.

Safety outcome

Safety outcomes of the study were the proportion of local and systemic adverse events that developed after the use of the product were assessed at day three and day seven. Information about adverse events (AEs) was documented, including the onset or reporting date, resolution date, severity level (ranging from mild to severe), any treatment given for the AE, and its final outcome (whether it was resolved, resolved with sequelae, ongoing, unknown, or fatal). AEs were classified as serious if they led to outcomes like death, hospitalization or extended hospital stay, congenital anomalies, permanent disability, or incapacity.

Statistical analysis

The outcome for quantitative variables were expressed as mean ± standard deviation. Student's t-test was performed with 95% confidence intervals with p-value <0.05 considered to be statistically significant. The qualitative variables were expressed as proportion and numbers.

## Results

From April 2016 to April 2023, a total of 2002 individuals were enrolled in this prospective, multi-centric, observational single-arm registry study to evaluate the clinical safety and effectiveness of the ointment containing heparin sodium and benzyl nicotinate in patients with thrombophlebitis. The mean age of the participants was 41.81±15.06 years, and more than half were male (58.15%), followed by females (41.7%) and transgenders (0.05%) (Table 3).

**Table 3:** Sociodemographic and clinical details of the participants (n=2002) SD: Standard deviation; IV: intravenous

Characteristics	Variables
Age (years), mean (SD)	41.81 (5.06)
Height (cm), mean (SD)	156.62 (24.42)
Weight (kg), mean (SD)	61.41 (13.34)
Gender	
Male, n (%)	1166 (58.15)
Female, n (%)	836 (41.85)
Region	
North, n (%)	319 (15.93)
West, n (%)	459 (22.93)
Central, n (%)	441 (22.03)
East, n (%)	340 (16.98)
South, n (%)	443 (22.13)
Speciality of Doctors	
General Practitioner, n (%)	596 (29.80)
General Surgeon, n (%)	543 (27.10)
Orthopaedic Surgeon, n (%)	424 (21.20)
General Consultants, n (%)	379 (18.90)
Gynaecologists, n (%)	60 (3.00)
Cause of thrombophlebitis	
IV Fluids, n (%)	1213 (60.58)
IV Drugs, n (%)	438 (21.89)
IV Fluids & IV Drugs Both, n (%)	217 (10.84)
Others, n (%)	134 (6.69)
<1 day, n (%)	3 (0.15)
1-3 days, n (%)	1099 (54.89)
4-7 days, n (%)	623 (31.12)
8-14 days, n (%)	431 (21.53)
>14 days, n (%)	6 (0.35)

The zone-wise break up of patients from all over India was 22.93% from the west, 22.13% from the south, 22.03% from the center, 16.98% from the east, and 15.93% from the north. The treating doctors belonged to different specialties with general practitioners (29.80%) being the most frequent, followed by general surgeons (27.10%), orthopedic surgeons (21.20%), consulting physicians (18.90%), and gynecologists (3%). (Table 3). All participants who had thrombophlebitis were included in the study, and it was noted that IV fluids were the leading cause of thrombophlebitis with 60.58%, followed by 21.89% for IV drugs. At the beginning of the study, thrombophlebitis was present in 54.89% of individuals for 1-3 days, 31.12% for 4-7 days and 21.53% for 8-14 days (Table 3).

Effectiveness outcome

Improvement in Phlebitis

**Figure 1:**
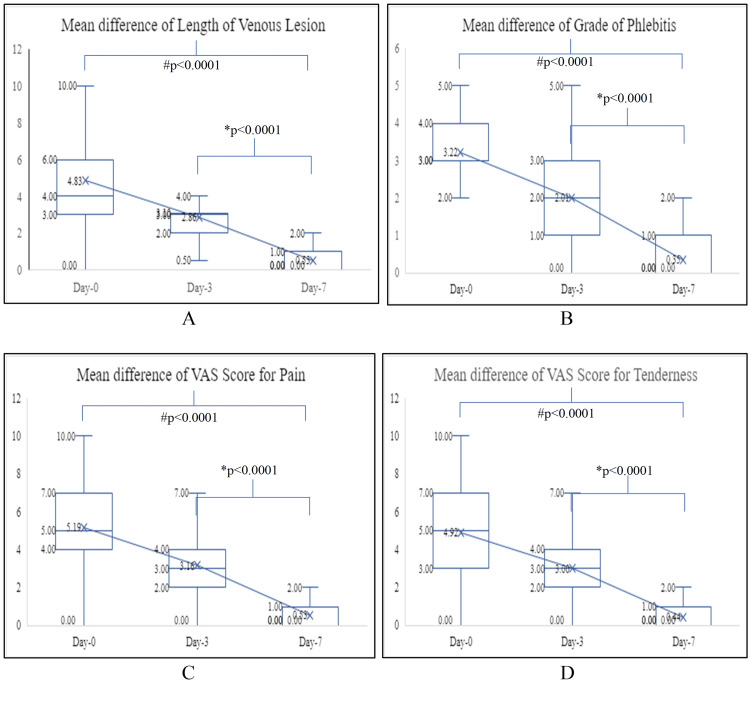
Scores for different variables at different times during the study period. # Signifies a statistically significant value between baseline and Day 3 & baseline and Day 7. *Signifies a statistically significant value between Day 3 and Day 7 VAS: Visual analog scale

At baseline, the mean grade of phlebitis was 3.22 ± 1.21. The grade of phlebitis fell significantly to 2.01 ± 1.04 on day three (p<0.001) and 1.35 ± 1.82 on day seven (p<0.0001). The grade of phlebitis significantly decreased from baseline to day three & day seven by 37.58% & 58.07% respectively. The improvement in the grade of phlebitis was statistically significant from day three to day seven with the mean grade falling by 32.84% (Table 4) (Figure 1).

Improvement in the Length of the Venous Lesion

At baseline, the mean length of the venous lesion was 4.83 ± 2.79 cm. The length of the venous lesion reduced significantly to 2.87 ± 1.71 cm on day three (p<0.001) and 0.53 ± 1.04 cm on day seven (p<0.0001). The length of venous lesion significantly decreased from baseline to day three & day seven by 40.8% & 89.02% respectively. Improvement in the length of the venous lesion was statistically significant from day three to day seven with mean length falling by 81.53% (Table 4) (Figure 1).

**Table 4:** Scores for different variables at different times during the study period (n=2002) VAS: Visual analog scale

Variables	Baseline	Day 3	Day 7	Baseline vs Day 3	Baseline vs Day 7	Day 3 vs Day 7
Grade of phlebitis	3.22 ± 1.21	2.01 ± 1.04	1.35 ± 1.82	p<0.001	p<0.0001	p<0.0001
Length of venous lesion (cm)	4.83 ± 2.79	2.87 ± 1.71	0.53 ± 1.04	p<0.001	p<0.0001	p<0.0001
VAS score for pain	5.17 ± 2.15	3.16 ± 1.91	1.31 ± 1.69	p<0.001	p<0.0001	p<0.0001
VAS score for tenderness	4.92 ± 2.24	2.99 ± 2.06	0.94 ± 1.56	p<0.001	p<0.0001	p<0.0001
Data was expressed as mean ± SD and for p values, a paired t-test was applied						

Improvement in Pain

At baseline, the mean VAS score for pain was 5.17 ± 2.15. The VAS score for pain fell significantly to 3.16 ± 1.91 on day three (p<0.001) and 1.31 ± 1.69 on day seven respectively (p<0.0001). The VAS score for pain significantly decreased from baseline to day three & day seven by 38.87%& 74.66% respectively. Improvement in the VAS score for pain was statistically significant from day three to day seven with the mean VAS score falling by 58.54% (Table 4) (Figure 1).

Improvement in Tenderness

At baseline, the mean VAS score for tenderness was 4.92 ± 2.24. The VAS score for tenderness fell significantly to 2.99 ± 2.06 on day three (p<0.001) and 0.94 ± 1.56 on day seven respectively (p<0.0001). The VAS score for tenderness significantly decreased from baseline to day three & day seven by 39.22%& 80.89% respectively. Improvement in the VAS score for tenderness was statistically significant from day three to day seven with the mean VAS score falling by 68.56% (Table 4) (Figure 1).

Safety outcome

Following the application of the ointment containing heparin sodium and benzyl nicotinate, the participants were assessed for local and systemic AEs. There were no local or systemic AEs at baseline, but very few participants, 0.25% on day three and 0.05% on day seven experienced local and systemic AEs. Redness (1.40%), itching (1.35%), rashes (0.55%), allergies (0.45%), burning at the site (0.40%), irritation (0.40%), pain at the local site (0.30%), vomiting (0.10%), and fever (0.05%) were the most frequent AEs. 3.9% of patients had mild AEs while 0.8% had moderate. 95.2% of patients experienced no AEs. Out of all these AEs, 57% were resolved in a single day, 34.35% in two days, and the remaining AEs were resolved in three days (Table 5).

**Table 5:** Distribution of participants according to nature of adverse events (n=2002)

Nature of Adverse events	Frequency	Percentage	Severity of adverse events	
Redness at application site	28	1.40%	Mild-23	Moderate-5
Itching at application site	27	1.35%	Mild-23	Moderate-4
Rashes at application site	11	0.55%	Mild-9	Moderate-2
Allergy at application site	9	0.45%	Mild-7	Moderate-2
Burning at application site	8	0.40%	Mild-3	Moderate-2
Irritation at application site	8	0.40%	Mild-8	
Swelling at the local site	6	0.30%	Mild-6	
Painful local site	4	0.20%	Mild-4	
Vomiting	2	0.10%	Mild-2	
Fever	1	0.05%	Mild-1	

Physician’s global assessment of tolerability and effectiveness

**Figure 2:**
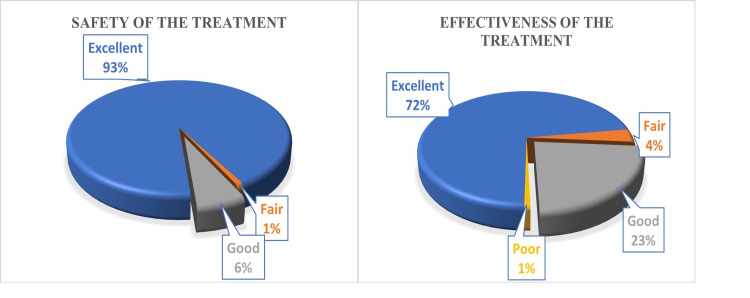
: Physician’s global assessment of effectiveness and tolerability (n=2002)

At the end of the study (Day seven), all participants were assessed for effectiveness and safety parameters based on the physician’s global assessment of tolerability and effectiveness. The results concluded that treatment effectiveness was excellent in 72% of patients, good in 23%, but fair or poor in 5% of patients, and treatment safety was excellent in 93% of patients, good in 6%, but fair in 1% of patients (Figure 2).

## Discussion

One of the most often performed invasive procedures in hospital-based treatment is IV cannulation and SVT is the most common complication for the same. Although thrombophlebitis is often a benign and self-limiting condition, it can cause severe erythema, swelling, pain, and discomfort around a superficial vein. Although local therapy may lessen the patient's discomfort and severe symptoms, it may not be able to stop complications, infections, or the spread of the clot into the deep venous system. To reduce the incidence of thrombophlebitis below 5%, preventive steps should be taken [9].

For the prevention and treatment of superficial thrombophlebitis, many heparin formulations have been investigated and commercially marketed [13]. The present study highlights that ointment containing 50 IU of heparin proves effectiveness of 72% and tolerance of 93% in the treatment of SVT.

Trials on topical heparin preparations have employed heparin application three times per day for a maximum of seven days or until the lesions heal for the management of SVT [16]. Although topical treatments are undoubtedly simpler to give, concurrent use of heparin infusion (5000 U) before and during the administration of antineoplastic drugs for a length of 12 hours reduces the occurrence of phlebitis in patients with ovarian cancer [17].

In conditions like superficial thrombophlebitis (inflammation or swelling of a vein due to a blood clot), hematoma (collection of blood outside the blood vessels, typically caused by an injury or trauma that damages blood vessels), or other localized inflammatory conditions, it is used to treat the relief from pain, inflammation, and improvement of local blood circulation [12]. In the study, it was found that in 72% of patients, treatment effectiveness was excellent, and in 93% of patients, treatment safety was excellent.

When no prophylaxis is given, prior research done by Saji et al., 2015 indicates a 50% overall incidence risk of thrombophlebitis, with 61% getting Grade 1 phlebitis and 39% having Grade 2 phlebitis [18]. In this study, the mean grade of phlebitis was more than three at the beginning of therapy but was much lower (around one) after the application of heparin sodium and benzyl nicotinate ointment for seven days. Additionally, it was observed that on days three and seven, respectively, the mean length of the venous lesion VAS score for pain and tenderness, all significantly decreased. This suggests that with the use of ointment containing Heparin sodium and Benzyl Nicotinate, the severity of phlebitis, length of the venous lesion, and VAS score for pain and tenderness were all significantly lower than baseline.

The usage of heparin sodium and benzyl nicotinate ointment has been linked in the literature to adverse effects such as rash, urticaria, and redness [13]. However, it was established in the present study that 95.2% of patients experienced no AEs. AEs were mild in 3.9% of cases and serious in 0.8% of cases. Most people who had AEs said that they were resolved in a day or less.

The present study has its limitations too, such as a lack of a control group and the use of subjective outcome measures (VAS score). However, despite this, the study provides a valuable foundation for future research. It highlights the potential of this treatment as a promising option for SVT management.

## Conclusions

SVT is recognized as a major complication of IV cannulation. The clinical effectiveness of heparin sodium and benzyl nicotinate ointment was significantly increased on days three and seven compared to baseline. The combination was well tolerated with non-serious side effects that developed in less than 1% of participants and most of them were resolved within a day. Thus, the prospective, observational, single-arm registry study established heparin sodium and benzyl nicotinate ointment (Thrombophob Ointment) was effective and well tolerated for the treatment of thrombophlebitis in Indian patients.
